# Efficacy and Safety of Faecal Microbiota Transplantation for Acute Pancreatitis: A Randomised, Controlled Study

**DOI:** 10.3389/fmed.2021.772454

**Published:** 2022-01-10

**Authors:** Ling Ding, Cong He, Xueyang Li, Xin Huang, Yupeng Lei, Huajing Ke, Hongyan Chen, Qinyu Yang, Yan Cai, Yuanhang Liao, Wenhua He, Liang Xia, Huifang Xiong, Nonghua Lu, Yin Zhu

**Affiliations:** Department of Gastroenterology, The First Affiliated Hospital of Nanchang University, Nanchang, China

**Keywords:** acute pancreatitis, faecal microbiota transplantation, gut microbiota dysbiosis, gastrointestinal dysfunction, infected pancreatic necrosis

## Abstract

**Aims:** We investigated whether faecal microbiota transplantation (FMT) decreases intra-abdominal pressure (IAP) and improves gastrointestinal (GI) dysfunction and infectious complications in acute pancreatitis (AP).

**Methods:** In this first randomised, single-blind, parallel-group, controlled study, we recruited and enrolled consecutive patients with AP complicated with GI dysfunction. Eligible participants were randomly assigned to receive faecal transplant (*n* = 30) or normal saline (*n* = 30) via a nasoduodenal tube once and then again 2 days later. The primary endpoint was the rate of IAP decline; secondary endpoints were GI function, infectious complications, organ failure, hospital stay and mortality. Analyses were based on intention to treat.

**Results:** We enrolled 60 participants and randomly assigned them to the FMT (*n* = 30) or control (*n* = 30) group. Baseline characteristics and disease severity were similar for both groups. IAP decreased significantly 1 week after intervention in both groups, with no difference in the IAP decline rate between FMT and Control group [0.1 (−0.6, 0.5) vs. 0.2 (−0.2, 0.6); *P* = 0.27]. Normal gastrointestinal failure (GIF) scores were achieved in 12 (40%) patients in the FMT group and 14 (47%) in the control group, with no significant difference (*P* = 0.60). However, D-lactate was significantly elevated in the FMT group compared to the control group, as calculated by the rate of decline [−0.3 (−3.7, 0.8) vs. 0.4 (−1.1, 0.9); *P* = 0.01]. Infectious complications occurred in 15 (50%) and 16 (53.33%) patients in the FMT and control groups, respectively (*P* = 0.80). However, interleukin-6 (IL-6) was significantly elevated in the FMT group compared to the control group, as calculated by the rate of decline [0.4 (−3.6, 0.9) vs. 0.8 (−1.7, 1.0); *P* = 0.03]. One participant experienced transient nausea immediately after FMT, but no serious adverse events were attributed to FMT.

**Conclusion:** FMT had no obvious effect on IAP and infectious complications in AP patients, though GI barrier indictors might be adversely affected. Further multi-centre studies are needed to confirm our findings. The study was registered at https://clinicaltrials.gov (NCT02318134).

## Introduction

Acute pancreatitis (AP) is an acute inflammatory disease of the pancreas. The incidence of AP ranges from 5 to 30 cases per 100,000, with an overall case fatality rate of 5% (1, 2). Approximately 80% of AP cases are mild and self-limited, and ~20% of patients have a severe disease course with persistent organ failure (OF) and/or infected pancreatic necrosis (IPN), with a mortality risk as high as 20~30% (3). The gastrointestinal (GI) tract is considered not only a target organ during systemic inflammatory response syndrome (SIRS) and multiple organ dysfunction syndrome (MODS) but also a “motor organ” of gut-derived infection (4, 5). Moreover, GI dysfunction has been proven to be associated with adverse outcomes in AP (6). Although the mechanisms underlying GI dysfunction in the early phase of AP are complicated, early GI barrier dysfunction is considered to be the main cause (7). In general, GI microbiota dysbiosis induces injury to the biological barrier, which plays a key role in the pathogenesis of gut-driven infection. Therefore, maintenance of the GI microecology balance is potentially an effective method for treating GI dysfunction and gut-driven infection and improving clinical outcomes in AP.

Several treatments targeting GI microflora dysbiosis have been studied, including antibiotics, probiotics, and selective decontaminants of the digestive tract, among others (4). Probiotics, as an adjunct to enteral nutrition, have long been investigated as a measure to improve intestinal barrier dysfunction and prevent secondary infection in AP. Indeed, several clinical studies have assessed the effect of probiotic prophylaxis, though with contradictory results, and significant heterogeneity between trials with regard to the type, dose and treatment duration of probiotics was noted (8, 9). For example, a multi-centre, double-blind, placebo-controlled trial failed to show a beneficial effect of probiotic prophylaxis on the occurrence of infectious complications, and mortality in the probiotics group was approximately twice as high as that in the placebo group (8). Such high mortality in those taking probiotics has been attributed to a lethal combination of mainly proteolytic pancreas enzymes and the probiotic therapy, and elevated levels of lactic acid produced by bacterial fermentation of carbohydrates are a key contributing factor (10). Nevertheless, data thus far are not sufficient to draw a conclusion regarding the effects of probiotics on patients with predicted severe acute pancreatitis (SAP). Consequently, there is a clear need for other innovative strategies.

Worldwide, interest in faecal microbiota transplantation (FMT) as an “ecological” therapy for several diseases is growing rapidly, representing a more comprehensive approach to microbiota restoration. FMT consists of administering faecal material from a healthy donor into the intestinal tract of a patient. Unlike the few bacterial strains included in probiotics, FMT material includes practically all the bacteria, viruses, eukaryotes, and metabolites from a healthy donor. By restoring the intestinal microbiota balance, FMT has shown excellent effects on recurrent *Clostridioides difficile* infection (11, 12). Additionally, patients with SIRS and severe diarrhoea of unclear aetiology who are persistently unresponsive to broad-spectrum antibiotics reportedly improve rapidly with FMT in the intensive care unit (ICU) (13). Improvements in clinical parameters have also been associated with shifts in recipients' microbiota patterns towards those of donors, further attributing patient recovery. FMT may therefore constitute a treatment for AP patients with GI dysfunction, yet no randomised controlled study evaluating FMT in AP patients has been published to date.

Here, we present the first randomised, single-blind, parallel-group, controlled study to clarify the efficacy and safety of FMT on declining intra-abdominal pressure (IAP), GI dysfunction improvement, and infectious complications in patients with AP.

## Patients and Methods

### Study Design and Participants

We conducted a randomised, single-blind, parallel-group, controlled, single-centre study in consecutive adults diagnosed with AP complicated with GI dysfunction in the setting of the ICU. Eligible participants were randomised 1:1 to receive faecal transplant or normal saline (NS) via a nasoduodenal tube twice (once every 2 days). The primary end point was the rate of IAP decline at 1 week after intervention. This study was investigator-initiated and investigator-driven and performed in accordance with the principles of the Revised Declaration of Helsinki. Written informed consent was obtained from all participants or their legal representatives following protocols approved by the institutional review boards of the First Affiliated Hospital of Nanchang University (No. 2014032). The study was registered at https://clinicaltrials.gov (NCT02318134).

Adult patients with AP (aged 18–70 years) were recruited between November 2017 and April 2019 from the ICU, Department of Gastroenterology, First Affiliated Hospital of Nanchang University. The diagnosis of AP requires two of the following three features: (1) upper abdominal pain; (2) serum lipase or amylase activity at least three times greater than the upper limit of normal; and (3) characteristic findings of AP on contrast-enhanced computed tomography (CECT) (14). Participants were eligible only if their AP was complicated with GI dysfunction, which was defined as intra-abdominal hypertension (IAH) and GI symptoms or signs including obvious abdominal distention, abdominal rumbling sound weakening or disappearance and no self-defecation (15). The time from AP onset to enrolment was restricted to two weeks. For safety reasons, we did not include participants with GI haemorrhage or GI fistula. Patients with multiple organ failure (MOF) were also excluded (16). Patients were not eligible if they had diabetes and autoimmune diseases or were pregnant or lactating.

### Donors and Sample Preparation

Faecal donors were recruited for this study from among students at Nanchang University. Once recruited, the donors were instructed to maintain a healthy lifestyle during the collection period. Prospective donors underwent a medical interview, and laboratory testing was performed within 1 month before faecal donation. The donors were screened according to guidelines (17) and were recruited according to pre-planned inclusion criteria determined with standard screening methods (shown in Supplementary Material 1).

Volunteers who met the selection criteria donated stool samples on the day of a patient's FMT. The donors were provided with a clean container. Immediately after producing the sample, the donors were instructed to deliver it to our facility within 30 min. Thereafter, the sample was processed as soon as possible. Each FMT material was prepared freshly from one single donor. Fresh stool (50 g) was diluted with 200 ml sterile saline and mixed in a blender. The homogenised solution was filtered twice through a pre-sterilised metal sieve. The sample was centrifuged (3000 rpm, 5 min), and the precipitate was dissolved in 200 mL NS twice. In this way, we obtained a more concentrated product. All preparation procedures were performed in a biological safety cabinet. The final filtrate (200 mL) or NS was drawn into a 50-mL sterile sealed syringe and infused into each patient via a nasoduodenal tube.

### Randomisation

A randomisation sequence for 60 participants with an allocation ratio of 1:1 was generated using SPSS by an independent statistician who was not involved in the clinical execution of the trial. The method of allocation concealment was the sequentially numbered sealed opaque envelope technique. Each participant was assigned a study number at enrolment. An allocator (non-study personnel) then divided the participants into the FMT or control group based on the randomisation sequence; the process was carried out in a closed room, and the allocation sequence was immediately disposed of. The randomisation key was revealed to researchers when all participants completed the 6-month follow-up and the data analysis had been completed.

### Interventions

All participants underwent interventions 1 day after allocation. In the FMT group, participants received 200 mL fresh donor faeces twice (once every 2 days). In the control group, participants received 200 mL NS. As mentioned above, the faecal transplant material or NS was drawn into a 50-mL sterile sealed syringe and infused via a nasoduodenal tube, which was inserted into the distal duodenum by endoscopy. The procedure was performed within 5 min at the bedside of the pancreatic ICU by a researcher who was not involved in the clinical execution of the trial. The patients were blinded to the identity of the intervention that they were receiving because both the FMT material and NS were stored in sealed syringes. Before FMT or NS, laxative was stopped for at least 1 h to retain stool. To ensure maximum delivery and colonisation, we first ensured at least a 4-h gap between administration of the study material and antibiotics (if prescribed) or laxatives. No probiotics or lactulose was used during hospitalisation.

All participants received routine treatment at admission according to AP guidelines (18), including goal-oriented fluid resuscitation, enteral nutrition as early as possible, and organ support, as needed. Antibiotic prophylaxis was not given routinely. The use of antibiotics was recorded, irrespective of indication. As all patients enrolled had GI dysfunction, we used the traditional Chinese medicine rhubarb (and/or mirabilite) for all of them. Other purge measures, including GI decompression or mannitol via nasoduodenal tube, were implemented according to the patients' condition. Abdominal puncture and drainage were performed for those with abdominal effusion. The details of the above therapies are shown in Supplementary Table S1.

### Outcomes of Interest

The primary end point was the rate of IAP decline. IAP was measured by standardised methodology (preferably the transvesical method, with a maximal instillation of 25 mL of saline in the supine position, measured at the end of expiration, with zeroing at the level of the midaxillary line). Other indicators of GI function were also evaluated. The gastrointestinal failure (GIF) score was calculated according to the need for enteral feeding and IAP (19). FI was defined when applied enteral feeding appeared to be unsuccessful and was discontinued because of repeated or profuse vomiting, high gastric residuals, ileus, severe diarrhoea, abdominal pain, or distension. FI was not considered present if enteral feeding was electively not prescribed or withheld/interrupted due to procedures. IAH was present if IAP was found to be 12 mmHg or higher, as confirmed by at least two measurements taken 1–6 h apart (20). Gut barrier function was assessed by detecting serum D-amino acid oxidase (DAO), D-lactate and endotoxin using commercial kits (Cloud-Clone Corp, TX, USA for DAO, Abcam, Cambridge, UK for D-lactate and ELX800 (Bio Tek) for endotoxin).

Other outcomes were any infectious complication, OF, functional assessments of inflammatory indicators, hospital stay and mortality. Infectious complications included IPN, infected ascites, bacteraemia, pneumonia, and urinary tract infection. Microbiological data for each infectious complication were collected. OF was defined as a score of 2 or more using the modified Marshall scoring system, as recommended by the 2012 Atlanta Classification Creation (14). Persistent OF was considered to be OF >48 h (16). Blood samples were collected from all included patients before and at 1 week after intervention; C-reactive protein (CRP) and procalcitonin (PCT) were measured using IMMAGE 800 (Beckman Coulter) and AFIAS-50 (JOINSTAR) equipment, respectively, and TNF-α and IL-6 were assessed using commercial kits (Cloud-Clone Corp, TX, USA). Definitions of the endpoints are detailed in Supplementary Table S2.

Patients were followed during their hospital stay. There was one follow-up visit at 6 months after discharge by clinical visit or telephone visit to assess readmission, mortality and adverse events. Data collection was prospectively input by a researcher who completed standardised case report forms. During the study, an independent data monitor checked the individual patients' data against the primary source data. After a double check of key variables by two researchers, data were exported unedited for statistical analyses.

### Statistical Analysis

Statistical analysis followed the intention-to-treat approach, and the results are reported according to CONSORT statement recommendations.

A sample size calculation was performed before initiating the trial. Using a preliminary trial of 10 participants, we determined 85% power for the FMT group and 50% power for the control group. Using a type I error of 0.05 and a type II error of 0.20 (80% power), we calculated that a minimal sample size of 48 with 24 patients in each group was required. To allow for dropouts, we included 60 participants, with 30 in each group.

The rate of decline in IAP, gut barrier and inflammatory indictors was calculated by (value before intervention – value 1 week after intervention)/value before intervention. We estimated relative risks (RRs) and 95% confidence intervals (CIs) for secondary outcomes. A two-tailed *P*-value < 0.05 was considered statistically significant. Data were analysed using SPSS software (v20.0; SPSS Inc., Chicago, IL, USA). An independent statistician who was not involved in the clinical execution of the trial performed interim analysis of efficacy and safety after the 40 participants had passed the 6-month follow-up and found no reason to terminate the study protocol due to serious adverse events due to FMT. The trial was finished as planned.

## Results

### Patient Characteristics

**Figure 1:**
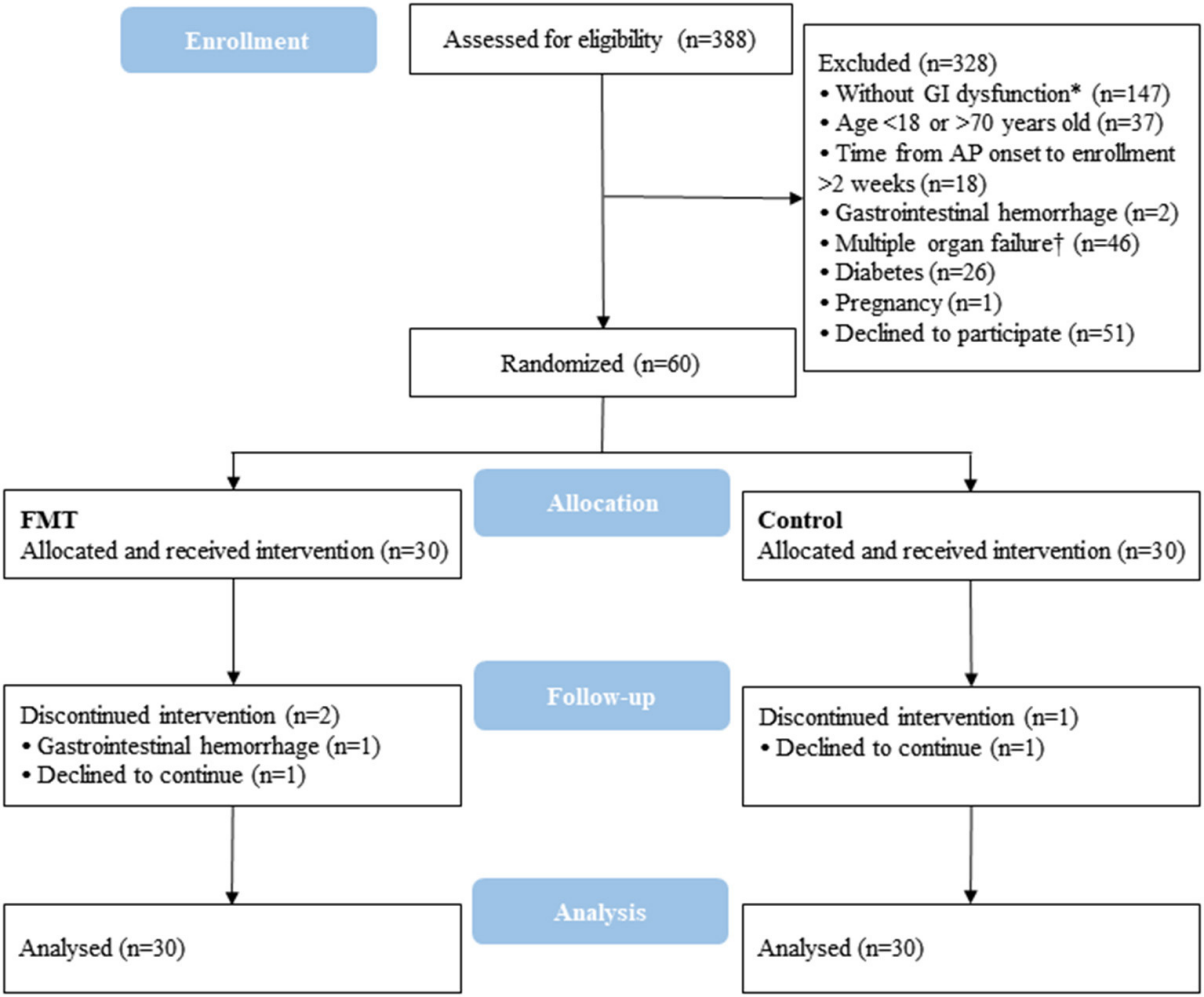
Flow chart of patients included and excluded from analysis according to CONSORT 2010. CONSORT, Consolidated Standards of Reporting Trials; GI, gastrointestinal; AP, acute pancreatitis; FMT, faecal microbiota transplantation. *GI dysfunction was defined as intra-abdominal hypertension and GI symptoms or signs including obvious abdominal distention, abdominal rumbling sound weakening or disappearance and no self-defecation. †Multiple organ failure was defined as two or more organ failures of the respiratory, cardiovascular and renal systems, which were defined according to the 2012 Atlanta Classification criterion.

Overall, between November 2017 and April 2019, 388 consecutive patients were assessed for eligibility; 328 were excluded for not having GI dysfunction (*n* = 147), age <18 or >70 years old (*n* = 37), time from AP onset to enrolment >2 weeks (*n* = 18), GI haemorrhage (*n* = 2), multiple organ failure (*n* = 46), diabetes (*n* = 26), pregnancy (*n* = 1), or declining to participate (*n* = 51). Ultimately, 60 patients were randomised and allocated 1:1 to the FMT group and control group. Three patients dropped out: two for GI haemorrhage or unwillingness in the FMT group and one for patient unwillingness in the control group. All analyses were performed on the basis of the intention-to-treat principle (Figure 1).

The mean age was 48.6 ± 13.0 years old, and there were 41 (68%) males and 19 (32%) females. The main cause of AP was hypertriglyceridaemia pancreatitis (*n* = 25, 42%), followed by biliary pancreatitis (*n* = 22, 37%) and alcoholic pancreatitis (*n* = 9, 15%). Most patients developed organ failure before intervention, with respiratory failure in 42 (70%), renal failure in 5 (8%), and circulatory failure in 2 (3%); 41 (68%) patients developed necrotizing pancreatitis. The FMT and control groups were comparable (*P* > 0.05) for all baseline characteristics, including sex, age, BMI, cause of AP, Charlson comorbidity index, severity scoring on admission, organ failure occurring before intervention, necrotizing pancreatitis, and time from admission to intervention (Table 1).

**Table 1:** Baseline characteristics of all participants.

	**All (***n*** = 60)**	**FMT (***n*** = 30)**	**Control** **(***n*** = 30)**	***P*** **-value**
Age, years	48.6 (13.0)	47.2 (12.4)	49.9 (13.7)	0.42
Sex				0.17
Male	41 (68%)	23 (77%)	18 (60%)	
Female	19 (32%)	7 (23%)	12 (40%)	
BMI, kg/m^2^	26.0 (3.9)	26.3 (3.7)	25.6 (4.2)	0.47
Cause of pancreatitis				0.38
Biliary	22 (37%)	9 (30%)	13 (43%)	
Alcohol	9 (15%)	6 (20%)	3 (10%)	
Hypertriglyceridemia	25 (42%)	14 (47%)	11 (37%)	
Other	4 (7%)	1 (3%)	3 (10%)	
Charlson comorbidity index, points	0 (0, 3)	0 (0, 2)	1 (0, 3)	0.27
Smoker	19 (32%)	10 (33%)	9 (30%)	0.78
Drinker	20 (33%)	12 (40%)	8 (27%)	0.27
APACHEII score, points*	8 (2, 24)	8 (2, 24)	8 (5, 18)	0.21
Modified Marshall score, points*	2 (0, 6)	2 (0, 6)	2 (0, 6)	0.83
SIRS score, points*	2 (0, 4)	2 (0, 4)	2 (0, 4)	0.62
Respiratory failure†	42 (70%)	24 (80%)	18 (60%)	0.09
Renal failure†	5 (8%)	2 (7%)	3 (10%)	0.99
Circulatory failure†	2 (3%)	0 (0%)	2 (7%)	0.47
Necrotizing pancreatitis	41 (68%)	20 (67%)	21 (70%)	0.78
IAP, mmHg	14.1 (2.5)	13.7 (2.4)	14.5 (2.5)	0.22
Time from symptoms onset to admission, days	3 (1.3, 4)	3 (1, 6)	3 (1, 9)	0.21
Time from admission to intervention, days	2 (1, 3.8)	2 (0, 11)	1.5 (1, 8)	0.15

*Data are given as the n (%), mean (standard deviation), or median (range). FMT, faecal microbiota transplantation; BMI, body mass index; APACHE II score, Acute Physiology and Chronic Health Evaluation II score; SIRS score, systemic inflammatory response syndrome score; IAP, intra-abdominal pressure*.

* *Scores assessed on admission*.

† *Organ failure determined before the day of intervention*.

### Intra-Abdominal Pressure

**Figure 2:**
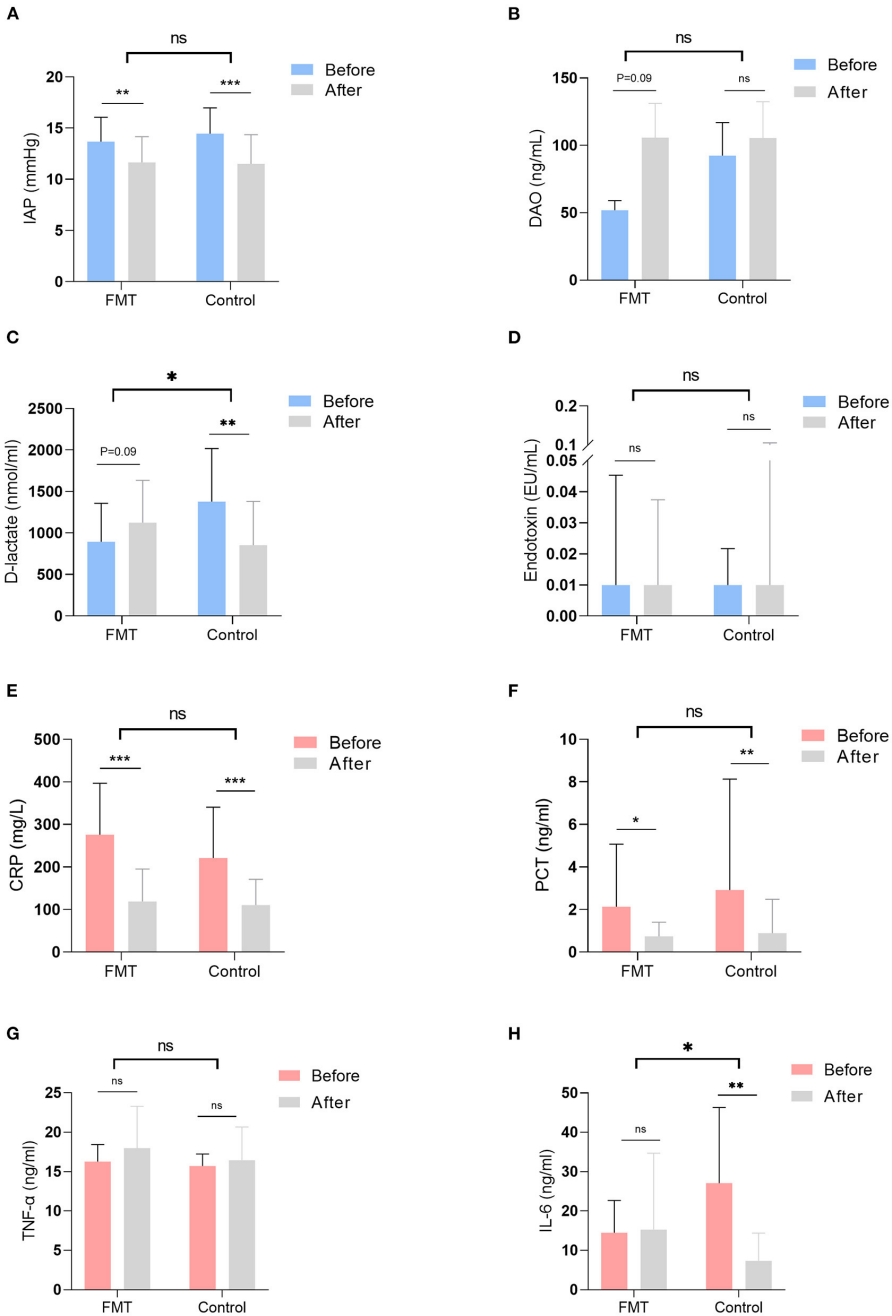
Gastrointestinal function and inflammatory indicators were examined and compared before and at 1 week after intervention in the two groups. The decline rate of those indicators was also compared between the FMT group and the control group. Gastrointestinal function indicators included IAP **(A)**, the level of DAO **(B)**, the level of D-lactate **(C)**, and the level of endotoxin **(D)**; inflammatory indictors included the level of CRP **(E)**, the level of PCT **(F)**, the level of THF-α **(G)**, and the level of IL-6 **(H)**. FMT, faecal microbiota transplantation; IAP, intra-abdominal pressure; DAO, D-amino acid oxidase; CRP, C-reactive protein; PCT, procalcitonin; TNF-α, tumour necrosis factor-α; IL-6, interleukin-6; ns, not significant. *P < 0.05, **P < 0.01, ***P < 0.001.

IAP was compared before and at 1 week after intervention in both groups, and the decline rates of AIP between the groups are shown in Table 2, Figure 2A. IAP decreased significantly at 1 week after intervention in both groups, with no difference in the decline rate between them [0.1 (−0.6, 0.5) vs. 0.2 (−0.2, 0.6); *P* = 0.27] (Figure 2A).

**Table 2:** IAP and gastrointestinal function compared between the FMT group and the control group.

	**All (***n*** = 60)**	**FMT (***n*** = 30)**	**Control** **(***n*** = 30)**	***P*** **-value**
Decline rate of IAP*	0.2 (−0.6, 0.6)	0.1 (−0.6, 0.5)	0.2 (−0.2, 0.6)	0.27
Normal GIF score†				0.60
Yes	26 (43%)	12 (40%)	14 (47%)	
No	34 (57%)	18 (60%)	16 (53%)	
Time required to achieve a normal GIF score	7 (1, 41)	7 (2, 32)	7.5 (1, 41)	0.94
GIF score, points	2 (0–3)	2 (0–3)	1.5 (0–3)	0.85
Enteral feeding <50% of calculated needs				0.99
Yes	22 (37%)	11 (37%)	11 (37%)	
No	38 (63%)	19 (63%)	19 (63%)	
FI				0.99
Yes	7 (12%)	3 (10%)	4 (13%)	
No	53 (88%)	27 (90%)	26 (87%)	
Large GRV				0.47
Yes	9 (15%)	3 (10%)	6 (20%)	
No	51 (85%)	27 (90%)	24 (80%)	

*Data are given as the n (%) or median (range). IAP, intra-abdominal pressure; FMT, faecal microbiota transplantation; GIF, gastrointestinal failure; FI, food intolerance; GRV, gastric residual volume*.

* *The decline rate of IAP was calculated by (value before intervention – value 1 week after intervention)/value before intervention*.

† *The normal GIF score means enteral feeding >50% of calculated needs, without FI and intra-abdominal hypertension. All indictors of gastrointestinal function were assessed at 1 week after intervention*.

### Gastrointestinal Function and Gastrointestinal Barrier Function

Normal GIF scores were achieved in 12 (40%) patients in the FMT group and 14 (47%) in the control group, with no significant difference (*P* = 0.60). Similarly, no significant differences (*P* > 0.05) were noted between the groups for other clinical GI variables, including the GIF score, enteral feeding <50% of calculated needs, food intolerance, and large gastric residual volume (GRV). Table 2 presents details for other GI functions.

Gut barrier indicators were compared before and at 1 week after intervention, and the decline rates of those indicators are shown in Table 3, Figures 2B–D. Serum D-amino acid oxidase (DAO) was elevated in the FMT group at 1 week after intervention, with no difference in the rate of decline between the groups [−0.2 (−46.2, 0.6) vs. 0.0 (−4.3, 0.8); *P* = 0.41] (Figure 2B). Although D-lactate was elevated in the FMT group at 1 week after intervention, it declined significantly in the control group (Figure 2C); D-lactate was elevated significantly in the FMT group compared to the control group, as calculated by the rate of decline [−0.3 (−3.7, 0.8) vs. 0.4 (−1.1, 0.9); *P* = 0.01] (Figure 2C). Conversely, no difference (*P* > 0.05) in the level of endotoxin was observed (Figure 2D).

**Table 3:** Gastrointestinal barrier function compared between the FMT group and the control group.

	**All** **(***n*** = 60)**	**FMT** **(***n*** = 30)**	**Control** **(***n*** = 30)**	***P*** **-value**
Decline rate of DAO*	−0.1 (−46.2, 0.8)	−0.2 (−46.2, 0.6)	0.0 (−4.3, 0.8)	0.41
Decline rate of D-lactate*	0.1 (−3.7, 0.9)	−0.3 (−3.7, 0.8)	0.4 (−1.1, 0.9)	**0.01**
Decline rate of endotoxin*	0.0 (−482.2, 1.0)	0.0 (−482.2, 1.0)	0.0 (−349.3, 1.0)	0.35

*Data are given as the median (range). FMT, faecal microbiota transplantation; DAO, D-amino acid oxidase*.

* *The gastrointestinal barrier indictors referred to the rate of decline calculated by (value before intervention – value 1 week after intervention)/value before intervention. P < 0.05 were bolded*.

### Infectious Complications

Table 4 shows the results for infectious complications and inflammatory indicators. There was no significant difference in the occurrence of any infectious complication between the FMT group and the control group (50% vs. 53%; *P* = 0.80), nor were there any significant differences between the groups in individual components and different infectious locations (*P* > 0.05). The RR for any infectious complication was 0.94 (95% CI, 0.57–1.53). The main cultured pathogens were gram-negative bacteria, including *Escherichia* (*n* = 5, 8%), *Enterobacter* (*n* = 7, 12%), *Klebsiella* (*n* = 7, 12%), *Acinetobacter* (*n* = 11, 18%), *Pseudomonas* (*n* = 3, 5%), and others (*n* = 3, 5%), followed by gram-positive bacteria, including *Staphylococcus* (*n* = 10, 17%), *Enterococcus* (*n* = 9, 15%), and others (*n* = 2, 3%). Twenty percent of the patients had fungal infections, including *Candida*.

**Table 4:** Infectious complications compared between the FMT group and the control group.

	**All (***n*** = 60)**	**FMT (***n*** = 30)**	**Control** **(***n*** = 30)**	***P*** **-value**
Any infectious complications	31 (52%)	15 (50%)	16 (53%)	0.80
Documented IPN	12 (20%)	4 (13%)	8 (27%)	0.20
Suspected or documented IPN	19 (32%)	10 (33%)	9 (30%)	0.78
Infected ascites	4 (7%)	2 (7%)	2 (7%)	0.99
Bacteraemia	19 (32%)	9 (30%)	10 (33%)	0.78
Pneumonia	14 (23%)	6 (20%)	8 (27%)	0.54
Urinary tract infection	10 (17%)	3 (10%)	7 (23%)	0.17
Gram-negative bacteria				
*Escherichia*	5 (8%)	4 (13%)	1 (3%)	0.35
*Enterobacter*	7 (12%)	2 (7%)	5 (17%)	0.42
*Klebsiella*	7 (12%)	3 (10%)	4 (13%)	0.99
*Acinetobacter*	11 (18%)	5 (17%)	6 (20%)	0.74
*Pseudomonas*	3 (5%)	1 (3%)	2 (7%)	0.99
Other gram-negative bacteria*	3 (5%)	1 (3%)	2 (7%)	0.99
Gram-positive bacteria				
*Staphylococcus*	10 (17%)	4 (13%)	6 (20%)	0.49
*Enterococcus*	9 (15%)	4 (13%)	5 (17%)	0.99
Other gram-positive bacteria†	2 (3%)	1 (3%)	1 (3%)	0.99
Fungi				
*Candida*	12 (20%)	4 (13%)	8 (27%)	0.33
Multidrug-resistant bacteria	24 (40%)	12 (40%)	12 (40%)	0.99
CRE	10 (17%)	4 (13%)	6 (20%)	0.49
CRAB	7 (12%)	4 (13%)	3 (10%)	0.99
CRPAE	1 (2%)	0 (0%)	1 (3%)	0.99
MRS	9 (15%)	3 (10%)	6 (20%)	0.47
Decline rate of CRP‡	0.6 (−5.4, 0.9)	0.6 (−0.6, 0.9)	0.5 (−5.4, 0.9)	0.72
Decline rate of PCT‡	0.6 (−13.8, 1.0)	0.6 (−6.5, 0.9)	0.7 (−13.8, 1.0)	0.46
Decline rate of TNF-α‡	0.0 (−1.0, 0.2)	−0.1 (−1.0, 0.2)	0.0 (−0.9, 0.2)	0.29
Decline rate of IL-6‡	0.7 (−3.6, 1.0)	0.4 (−3.6, 0.9)	0.8 (−1.7, 1.0)	**0.03**

*Pathogens isolated from 31 patients with any infectious complications are listed at the genus level. If the same organism was cultured from the same sites at different times for one patient, this organism is listed only once. Data are given as the n (%) or median (range). FMT, faecal microbiota transplantation; IPN, infectious pancreatic necrosis; CRE, carbapenem-resistant Enterobacteriaceae; CRAB, carbapenem-resistant Acinetobacter baumannii; CRPAE, carbapenem-resistant Pseudomonas aeruginosa; MRS, methicillin-resistant Staphylococcus; CRP, C-reactive protein; PCT, procalcitonin; TNF-α, tumour necrosis factor-α; IL-6, interleukin-6*.

* *Rapidly growing mycobacteria (1) and Serratia marcescens (2)*.

† *Unknown (1)*.

‡ *Inflammatory indictors referred to the rate of decline calculated by (value before intervention – value 1 week after intervention)/value before intervention. P < 0.05 were bolded*.

Inflammatory indicators were compared before and at 1 week after intervention; the decline rates of those indicators between the two groups are shown in Table 4, Figures 2E–H. In both groups, C-reactive protein (CRP) and procalcitonin (PCT) declined significantly at 1 week after intervention, with no difference in the decline rate between the groups [CRP, 0.6 (−0.6, 0.9) vs. 0.5 (−5.4, 0.9); *P* = 0.72; PCT, 0.6 (−6.5, 0.9) vs. 0.7 (−13.8, 1.0); *P* = 0.46] (Figures 2E,F). No difference (*P* > 0.05) was observed in the level of TNF-α (Figure 2G). IL-6 was not different before and after intervention in the FMT group, though it declined significantly in the control group (Figure 2H). IL-6 was significantly elevated in the FMT group compared to the control group, as calculated by the rate of decline [0.4 (−3.6, 0.9) vs. 0.8 (−1.7, 1.0); *P* = 0.03] (Figure 2H).

### Other Clinical Outcomes

Complications were not significant between the groups, including any persistent OF (80% vs. 77%; *P* = 0.75), enterocutaneous fistula (3% vs. 3%; *P* = 0.99), abdominal bleeding (7% vs. 0%; *P* = 0.49), GI bleeding (7% vs. 3%; *P* = 0.99), and GI perforation (0% vs. 1%; *P* = 0.99). Seven patients died due to multiple OF or infectious complications, 3 in the FMT group and 4 in the control group. Four patients died within 2 weeks of admission and 3 within 4 weeks of admission (Table 5).

**Table 5:** Other clinical outcomes compared between the FMT group and the control group.

	**All (***n*** = 60)**	**FMT (***n*** = 30)**	**Control** **(***n*** = 30)**	***P*** **-value**
Any persistent organ failure*	47 (78%)	24 (80%)	23 (77%)	0.75
Persistent respiratory failure	45 (75%)	24 (80%)	21 (70%)	0.37
Persistent renal failure	8 (13%)	3 (10%)	5 (17%)	0.70
Persistent circulatory failure	20 (33%)	9 (30%)	11 (37%)	0.58
Enterocutaneous fistula	2 (3%)	1 (3%)	1 (3%)	0.99
Abdominal bleeding	2 (3%)	2 (7%)	0 (0%)	0.49
Gastrointestinal bleeding	3 (5%)	2 (7%)	1 (3%)	0.99
Gastrointestinal perforation	1 (2%)	0 (0%)	1 (3%)	0.99
Severity according to 2012 Atlanta Classification				0.75
SAP	47 (78%)	24 (80%)	23 (77%)	
MSAP	13 (22%)	6 (20%)	7 (23%)	
Percutaneous or endoscopic transmural drainage	21 (35%)	10 (33%)	11 (37%)	0.79
Percutaneous or endoscopic transmural necrosectomy	13 (22%)	6 (20%)	7 (23%)	0.75
Open surgery	2 (3%)	1 (3%)	1 (3%)	0.99
Mechanical ventilation	22 (37%)	12 (40%)	10 (33%)	0.59
Renal replacement therapy	5 (8%)	2 (7%)	3 (10%)	0.99
Intensive care stay, days	11 (2, 103)	9 (3, 103)	11 (2, 43)	0.73
Hospital stay, days	21 (5, 125)	18.5 (9, 122)	23 (5, 125)	0.68
Mortality	7 (12%)	3 (10%)	4 (13%)	0.99

*Data are given as the n (%) or median (range). FMT, faecal microbiota transplantation; SAP, severe acute pancreatitis; MSAP, moderately severe acute pancreatitis*.

* *Patients with organ failure present at any time during admission, irrespective of the date of onset of organ failure, are included*.

No patients died during the 6-month follow-up, but seven patients needed readmission for different reasons: four patients (three in the control group and one in the FMT group) due to the complications of walled-off necrosis, all of whom were discharged after conservative treatment; two patients (one in each group) due to infected walled-off necrosis, all of whom were discharged after necrosectomy; and one patient in the FMT group due to ketoacidosis.

### Adverse Events

One participant had transient nausea immediately after FMT (probably due to FMT), and the patient declined the second FMT. No serious adverse events were attributed to FMT.

## Discussion

To the best of our knowledge, this is the first randomised controlled study to evaluate the efficacy and safety of FMT in AP patients. We found that FMT had no obvious effect on IAP or infectious complications in AP patients but that GI barrier indictors might be adversely affected. No serious adverse events were observed in patients with AP who underwent FMT.

Research to date suggests a bidirectional relationship between the gut microbiome and the severity of AP. Multiple factors strongly contribute to intestinal dysbiosis, including abnormal bowel motility and microcirculation disturbance (4). Nonetheless, accumulating evidence indicates that gut dysfunction in patients with AP, which involves weakening of mobility and impairment of the epithelial barrier, may facilitate translocation of gut bacteria to peripheral organs or the circulatory system and result in infectious complications (4, 8, 11). We have reported bidirectional modulation between the gut microbiota and NLRP3 in the progression of AP, suggesting interplay of the host and microbiome during AP (21). In recurrent *Clostridioides difficile* infection, FMT has shown excellent effects by restoring the intestinal microbiota balance (11, 12). Thus, we conducted this RCT to explore the efficacy and safety of FMT on decreasing IAP and improving GI dysfunction and infectious complications in AP patients.

Overall, key clinical parameters, including IAP, infectious complications, and other clinical outcomes, were not significantly different between the FMT group and the control group. However, GI barrier function might be adversely affected by FMT, as indicated by increased D-lactate. Patient selection, the timing of FMT, routine treatment, donor selection, FMT manufacturing and administration route likely impact the outcomes of FMT (22). Before intervention, 49 (82%) patients developed OF, including respiratory failure, renal failure and circulatory failure. The severity of AP and OF may influence the effect of FMT. Evidence suggests that intestinal blood flow at the mucosal level is generally reduced in AP. In a severely ill patient in a phase of severe systemic inflammation or OF, the blood flow and oxygen supply are reduced critically, and severe local inflammation occur at the mucosal level (23). It has been reported that prophylactic probiotics are associated with increased bacterial translocation and enterocyte damage in patients with OF (24). In addition, as the gut microbiota might be dramatically altered by intestinal inflammation, a healthy microbiota transferred to an inflamed gut might be rapidly altered, thus limiting its potential therapeutic effect. Therefore, two FMTs may not be sufficient for flora reconstitution. Although antibiotic prophylaxis was strongly discouraged in our study, antibiotics were used in approximately two-thirds of the patients, even though only half of all patients had a documented infection. Antibiotics were sometimes started pre-emptively on the basis of clinical suspicion of infection before bacterial culture results became available. Despite no differences between the groups, the use of antibiotic and purge measures may have impacted the effects of FMT. We based our protocols for donor faecal collection, delivery and manufacturing on published guidelines (17). Notably, FMT success is partly dependent on the microbial diversity and composition of the stool donor (25), though the ideal approach to donor selection has yet to be defined (26). A nasoduodenal tube was chosen for delivery, as it was indicated that the small intestine is the main source of bacterial translocation in AP (27). From the point of cost-effectiveness, FMT contributed to shortening of ICU stay by 2 days, and 5 days of hospitalisation might be important for saving medical costs and social costs (28, 29). Further multi-centre studies are needed to confirm our findings.

However, limitations need to be taken into consideration. First, although one participant had transient nausea immediately after FMT (probably due to FMT), no serious adverse events could be attributed to FMT. Nevertheless, some potential serious adverse events could not be identified in patients with serious conditions and might occur due to intestinal mucosal barrier injury in patients with AP (30). Further studies are needed to improve the safety of FMT with regard to laboratory processes, such as washed microbiota transplantation (31, 32). Second, this was a single-centre study, and we excluded patients with multiple OFs, which likely limited the representativeness of the results. Neither the doctors nor the researchers were blinded, which might induce bias. Third, there was no objective, clinically relevant definition of complicated GI dysfunction; thus, we used IAP as the primary endpoint to assess gut function, which may not reflect all aspects of gut function. However, we analysed different clinical indicators and gut barrier indicators to comprehensively assess gut function.

## Data Availability Statement

The datasets presented in this study can be found in online repositories. The names of the repository/repositories and accession number(s) can be found in the article/Supplementary Material.

## Ethics Statement

The studies involving human participants were reviewed and approved by the Institutional Review Boards of the First Affiliated Hospital of Nanchang University (No. 2014032). The patients/participants provided their written informed consent to participate in this study.

## Author Contributions

LD and CH designed and coordinated the study and drafted the manuscript. XL recruited the donors and performed the sample preparation and the FMT procedure. XH, YLe, and HK recruited the participants and performed routine treatment. HC and YC assisted in performing the FMT procedure. LD, QY, and YLi assisted in recording the CRF and measuring serum indictors. WH, LX, and HX performed the statistical analysis and interpreted the data. NL and YZ conceived of the idea, supervised the project, and revised the manuscript for important content. All authors read and approved the final manuscript.

## Funding

This work was supported by grants from the National Natural Science Foundation of China (NO. 81960128) and the Key Research and Development Program from the Science and Technology Department of Jiangxi Province (NO. 20192ACBL20037).

## Conflict of Interest

The authors declare that the research was conducted in the absence of any commercial or financial relationships that could be construed as a potential conflict of interest.

## Publisher's Note

All claims expressed in this article are solely those of the authors and do not necessarily represent those of their affiliated organizations, or those of the publisher, the editors and the reviewers. Any product that may be evaluated in this article, or claim that may be made by its manufacturer, is not guaranteed or endorsed by the publisher.
