# Supplementary material for: Efficacy and Safety of Faecal Microbiota Transplantation for Acute Pancreatitis: A Randomised, Controlled Study
Source: Front Med (Lausanne). 2022 Jan 10;8:772454. doi: 10.3389/fmed.2021.772454 (PMC8784600; doi:10.3389/fmed.2021.772454)
Supplement: Supplementary file 1 [file Table_1.DOCX]

*Supplementary Material*

# Supplementary Data

**Inclusion criteria for screening fecal donors:**

Inclusion criteria for screening fecal donors:

1. aged more than 18; years old;
2. previously and currently healthy;
3. normal weight (body mass index (BMI) between 18.5 and 24.9 kg/m^2^);
4. normal bowel movements.

Donor exclusion criteria included the following:

1. known or high risk of infectious diseases as HIV, HAV, HBV or HCV;
2. known diseases of digestive system such as inflammatory bowel disease, irritable bowel syndrome, chronic constipation or chronic diarrhea;
3. known systemic autoimmunity, metabolic, neurologic, malignancy diseases or atopic diseases;
4. use of antibiotics, probiotics, immunosuppressant, or other medication consumption in the past 6 months;
5. abuse of alcohol or drugs;
6. smoking;
7. tattoo or body piercing within the last 6 months;
8. family history of GI disorders, allergy, asthma, eczema, cardiovascular diseases, neurologic or mental illnesses;
9. participation in high risk sexual behaviours.

Blood routine, blood biochemistry, gastroscopy, and urea breath test were performed before the FMT donation. Donor blood was negative for common viruses (hepatitis A, B, and C, HIV, cytomegalovirus, Epstein-Barr, Herpes simplex, and Varicella zoster) and Treponema pallidum. Donor feces were negative for common enteric pathogens (Yersinia spp., Salmonella spp., Shigella spp., Campylobacter jejuni, C. difficile toxin, helminths, ova, parasites, rotavirus, amoeba, and Helicobacter pylori),.

# Supplementary Tables

Table S1. The details of therapy of all patients

|  | FMT (n=30) | Control (n=30) | P value |
| --- | --- | --- | --- |
| Antibiotics during FMT | 19 (63%) | 25 (83%) | 0.08 |
| Antibiotics* | 22 (73%) | 25 (83%) | 0.35 |
| Fluid resuscitation* | 30 (100%) | 30 (100%) | - |
| Enteral nutrition* | 30 (100%) | 30 (100%) | - |
| Traditional Chinese medicine*† | 30 (100%) | 30 (100%) | - |
| Mannitol via nasoduodenal tube* | 27 (90%) | 28 (93%) | 0.99 |
| Abdominal puncture and drainage* | 22 (73%) | 20 (67%) | 0.87 |
| Gastrointestinal decompression* | 19 (63%) | 20 (67%) | 0.51 |

Data are n (%). FMT, fecal microbiota transplantation. *The therapy based on patients’ symptoms and left to the treating clinicians’ discretion during admission. †The traditional Chinese medicine included rhubarb (and/or mirabilite).

Table S2. Definitions of endpoints

| endpoint | definitions | |
| --- | --- | --- |
| GIF score | points | Clinical symptomatology |
|  | 0 | Normal gastrointestinal function |
|  | 1 | Enteral feeding <50% of calculated needs or no feeding 3 days after abdominal surgery |
|  | 2 | Food intolerance (enteral feeding not applicable due to high gastric aspirate volume, vomiting, bowel distension, or severe diarrhoea) or IAH |
|  | 3 | Food intolerance and IAH |
|  | 4 | Abdominal compartment syndrome |
| Food intolerance | when applied enteral feeding appeared to be unsuccessful and had to be discontinued because of repeated or profuse vomiting, high gastric residuals, ileus, severe diarrhea, abdominal pain, or distension. | |
| IAH | if the IAP was found to be 12 mmHg or higher, as confirmed by at least two measurements taken 1-6 h apart | |
| Infectious complications |  | |
| Documented IPN | Positive culture of peripancreatic fluid or pancreatic necrosis obtained by either fine-needle aspiration or during the first percutaneous or endoscopic drainage | |
| Suspected IPN | Persistent fever and clinical deterioration with purulent drainage fluid from percutaneous or endoscopic drainage, while other sources of infection were absent | |
| Infected ascites | bacteria or fungi detected in aspirate of intraperitoneal fluid | |
| Bacteremia | positive blood culture | |
| Pneumonia | Positive sputum or endotracheal culture | |
| Urinary tract infection | Positive urine culture | |
| Organ failure* |  | |
| Respiratory failure | PaO2/FiO2 below 300 | |
| Renal failure | Serum Creatinine over 170 μmol/l or 1.9 mg/dl | |
| Circulatory failure | Systolic blood pressure below 90 mm Hg, not fluid responsive or pH below 7.3 | |

GIF score, gastrointestinal failure score; IAH, intra-abdominal hypertension; IAP, intra-abdominal pressure; IPN, infected pancreatic necrosis. *organ failure was defined using the Modified Marshall scoring system.
